# The True Anti-Phthisis Unit

**Published:** 1913-09-27

**Authors:** 


### The Hospital

The Workers' Newspaper of Administrative Medicine and Institutional Life, Administration, National Insurance and Health.

No. 1432, Vol. LIV. SATURDAY, SEPTEMBER 27, 1913. PRICE ONE PENNY.

### THE TRUE ANTI-PHTHISIS UNIT.

This is an age in which verbal self-expression shows prudence and the greatest tenderness for the self-love of others. Accordingly the title of the subject for discussion at the recent conference of the National Association for the Prevention of Consumption was "The Need for the Co-ordination of Anti-tuberculosis Measures." But the subject itself, as in the animation of debate became evident, was much more like "The Managing Partner of the Anti-tuberculosis Firm." There appeared to be several candidates for this post, as follows: first, on Sir Robert Philip's well-supported recommendation, the tuberculosis dispensary; second, the Public Health Department; third, the out-patient hall of the largest local general hospital. To these three, although no speaker seems to have noticed it, must, for completeness, be added a fourth—namely, that home of acupuncture the tuberculin dispensary, municipal or otherwise. Which of these four agencies is going to furnish the medical man who will have the say in advising as to what shall, and what shall not, be done in fighting the tuberculosis of a neighbourhood?

Curiously enough, the word "sanatorium" has not yet been mentioned. And yet, if asked for an unofficial opinion, we might name as the best existing bodies in England for the actual control of tuberculosis work two little-known sanatoria. Both have a good many years' experience of phthisical patients, of examining and treating them, of their real needs, foibles, possibilities. Neither enjoys the support of populous areas; accordingly executive officials are few and unhampered by uninformed colleagues. They are also in earnest, for the pinch of necessity has made them cut their coat according to their cloth, made them quick to meet real needs and so secure custom from those with patients to send. Their medical advisers have no ambition to cater for that phantasmal person the consumptive who cannot afford the fees of a private institution, but whom the Insurance Act misses; or to figure only as consultant diagnosticians. They take a real, lively, and most practical interest in all the details of institutional treatment. Accordingly, and especially lately, with the chances that have been open, these institutions have gone ahead. With some co-operation with county medical officers of health, both their honorary medical directors would make admirable tuberculosis directors.

This last proviso brings us to one of the very few points in this matter which are axiomatic. The Public Health Department must be closely consulted. The anti-tuberculosis campaign is but a branch of Public Health work, and when it comes, as it must do very soon and constantly, to the question of the sinews of war, one technical department must apportion the amount of funds to be devoted to fighting each disease. Otherwise there would be the unedifying spectacle of medical men interested in tuberculosis competing for municipal money with medical men interested in summer diarrhœa, enteric fever, diphtheria, and so forth. In this respect, things seem well enough as they are. The medical officer of health has been given a special adjutant for tuberculosis, as he had already been given a special adjutant for educational work. All he has to do is to allow him a free (clinical) hand. But two things should be said, the one as to dispensaries and the other as to their officers. Laymen have an awkward way of asking for results, of looking at returns and death-rates "before and after." Well, Professor Pearson's finding is on record that Aberdeen, till just lately, with no tuberculosis dispensaries at all, can show a more rapid fall in the tuberculosis death-rate than Edinburgh itself, the headquarters of that movement. As to tuberculosis officers, the text is supplied by Professor Rist's remarks in the discussion under notice, when he animadverted on the scarcity of men who could really be considered as tuberculosis experts.

There is no doubt, to speak plain truth, that in many of the numerous recent appointments influence has overcome real experience and merit. With a few exceptions, these successful candidates cannot take much of a stand as phthisiologists. The few who can will make their own positions; people will become nervous of contradicting them; they will deservedly sway counsel.

Professor Rist's contention has a bearing too on the claims of out-patient physicians. Outside London there are few authorities on tuberculosis: the ordinary consultant, unlike Sir Robert Philip himself or the late Dr. Ransome, cannot spare one part of the field of internal medicines the special culture it requires. Besides, "whole-time," fixed-salary workers are what are really needed. Finally, as to the tuberculin dispensary, the view expressed in these columns has always been that although intensive tuberculinisation is still on its trial, yet the chances seemed against its realising all the therapeutic claims made for it in a certain quarter. In sum, the question must be left to settle itself in practice: the true working anti-phthisis unit is still in the melting-pot. As has been indicated, here one, here another, voice will be heeded most. Besides the great general interest, there is also, as we have before remarked, a technical and professional one. For the whole-time phthisiologist will be the first law-made medical specialist.

